# The risk of PD-L1 expression misclassification in triple-negative breast cancer

**DOI:** 10.1007/s10549-022-06630-3

**Published:** 2022-05-27

**Authors:** Shani Ben Dori, Asaf Aizic, Asia Zubkov, Shlomo Tsuriel, Edmond Sabo, Dov Hershkovitz

**Affiliations:** 1 B. Rappaport Faculty of Medicine, Technion-Israel Institute of Technology, Haifa, Israel; 2 Institute of Pathology, Tel-Aviv Sourasky Medical Center, Dafna 5, 6492601 Tel Aviv, Israel; 3 Institute of Pathology, Carmel Medical Center, Haifa, Israel; 4 Sackler Faculty of Medicine, Tel-Aviv University, Tel-Aviv, Israel

**Keywords:** Triple-negative breast cancer, PD-L1, Atezolizumab, Biopsy, Misclassification

## Abstract

**Purpose:**

Stratification of patients with triple-negative breast cancer (TNBC) for anti-PD-L1 therapy is based on PD-L1 expression in tumor biopsies. This study sought to evaluate the risk of PD-L1 misclassification.

**Methods:**

We conducted a high-resolution analysis on ten surgical specimens of TNBC. First, we determined PD-L1 expression pattern distribution via manual segmentation and measurement of 6666 microscopic clusters of positive PD-L1 immunohistochemical staining. Then, based on these results, we generated a computer model to calculate the effect of the positive PD-L1 fraction, aggregate size, and distribution of PD-L1 positive cells on the diagnostic accuracy.

**Results:**

Our computer-based model showed that larger aggregates of PD-L1 positive cells and smaller biopsy size were associated with higher fraction of false results (*P* < 0.001, *P* < 0.001, respectively). Additionally, our model showed a significant increase in error rate when the fraction of PD-L1 expression was close to the cut-off (error rate of 12.1%, 0.84%, and 0.65% for PD-L1 positivity of 0.5–1.5%, ≤ 0.5% ,and ≥ 1.5%, respectively, *P* < 0.0001). Interestingly, false positive results were significantly higher than false negative results (0.51–22.62%, with an average of 6.31% versus 0.11–11.36% with an average of 1.58% for false positive and false negative results, respectively, *P* < 0.05). Furthermore, heterogeneous tumors with different aggregate sizes in the same tumor, were associated with increased rate of false results in comparison to homogenous tumors (*P* < 0.001).

**Conclusion:**

Our model can be used to estimate the risk of PD-L1 misclassification in biopsies, with potential implications for treatment decisions.

**Supplementary Information:**

The online version contains supplementary material available at 10.1007/s10549-022-06630-3.

## Introduction

Triple-negative breast cancer (TNBC) accounts for 15–30% of breast cancer cases and is the most aggressive type of breast cancer with high rates of distant metastases and poor survival rates [1, 2]. It is defined by the lack of expression of estrogen receptor, progesterone receptor, and human epidermal growth factor receptor 2 (HER2); hence it is insensitive to endocrine treatment and targeted therapies [3].

Immunotherapy in the matter of immune checkpoint inhibitors (ICIs) such as monoclonal antibodies targeting program death-ligand 1 (PD-L1) and program cell death 1 (PD-1) have reformed the treatment for numerous cancer types and recently, the addition of Atezolizumab to chemotherapy was approved for metastatic and unresectable locally advanced TNBCs [4–7]. PD-L1 positive tumors are defined according to immunohistochemical testing by PD-L1 expression on tumor-infiltrating immune cells accounting for at least 1% of the tumor area [8, 9]. It should be noted that different therapies requires different threshold of PD-L1 positivity and evaluation of PD-L1 positivity in both immune cells and tumor cells. While pembrolizumab threshold is PD-L1 positivity of > 10% and based on combined score of both tumor and inflammatory cells, Atezolizumab threshold is PDL-1 positivity of > 1% in immune cells [10].

Analysis of PD-L1 expression may be performed on tissue samples obtained from resection or on a core needle biopsy from either primary or metastatic sites [11], and hence, a biopsy should represent the entire tumor accurately. However, there are potential limitations that may lead to inaccurate classification of PD-L1 status, such as relatively small specimens or heterogeneous expression of PD-L1 within the tumor. Our study sought to characterize PD-L1 expression in immune cells on triple-negative breast cancer tumors and, using a computer-based algorithm, to estimate the risk of PD-L1 status misclassification (false negative and false positive results) in biopsies.

## Methods

We used two complementary methods to evaluate the risk for false results in the analysis of PD-L1 in TNBC. First, we examined the pattern of PD-L1 expression using tumor surgical samples from patients with TNBC. Then, based on those PD-L1 patterns we used the MATLAB software to establish virtual samples, which were further evaluated to determine the risk of false PD-L1 status classification.

### Clinical samples

Tumor tissue specimens were collected retrospectively from ten patients with TNBC, who had undergone surgical resection at Tel-Aviv Sourasky Medical Center. The study protocol was approved by the local ethics committee.

### Immunohistochemical staining

Freshly cut, 4 micron slides were stained using the VENTANA PD-L1 (SP142) Assay (Ventana Medical Systems, Tucson, Arizona) according to manufacturer instructions. Stainings were performed on a Ventana BenchMark Ultra immuno stainer (Ventana Medical Systems, Tucson, Arizona). Normal tonsil tissue was used as a control for each case.

Slides were scanned using the Phillips UltraFast Scanners (Philips Digital Pathology Solutions, Best, the Netherlands) to obtain high-resolution whole slide digital images.

### Analysis of the clinical samples

The samples were analyzed according to VENTANA PD-L1 (SP142) assay. Thousands of stained immune cells as aggregates or single cells were manually captured on the digital slides assisted by QuPath software version 0.2.3. These detailed measurements along with total tumor area provided important information including PD-L1 expression percentage, number of stained immune cells, and the area and pattern of stained immune cells in each tumor.

### Computer-based model

**Fig. 1:**
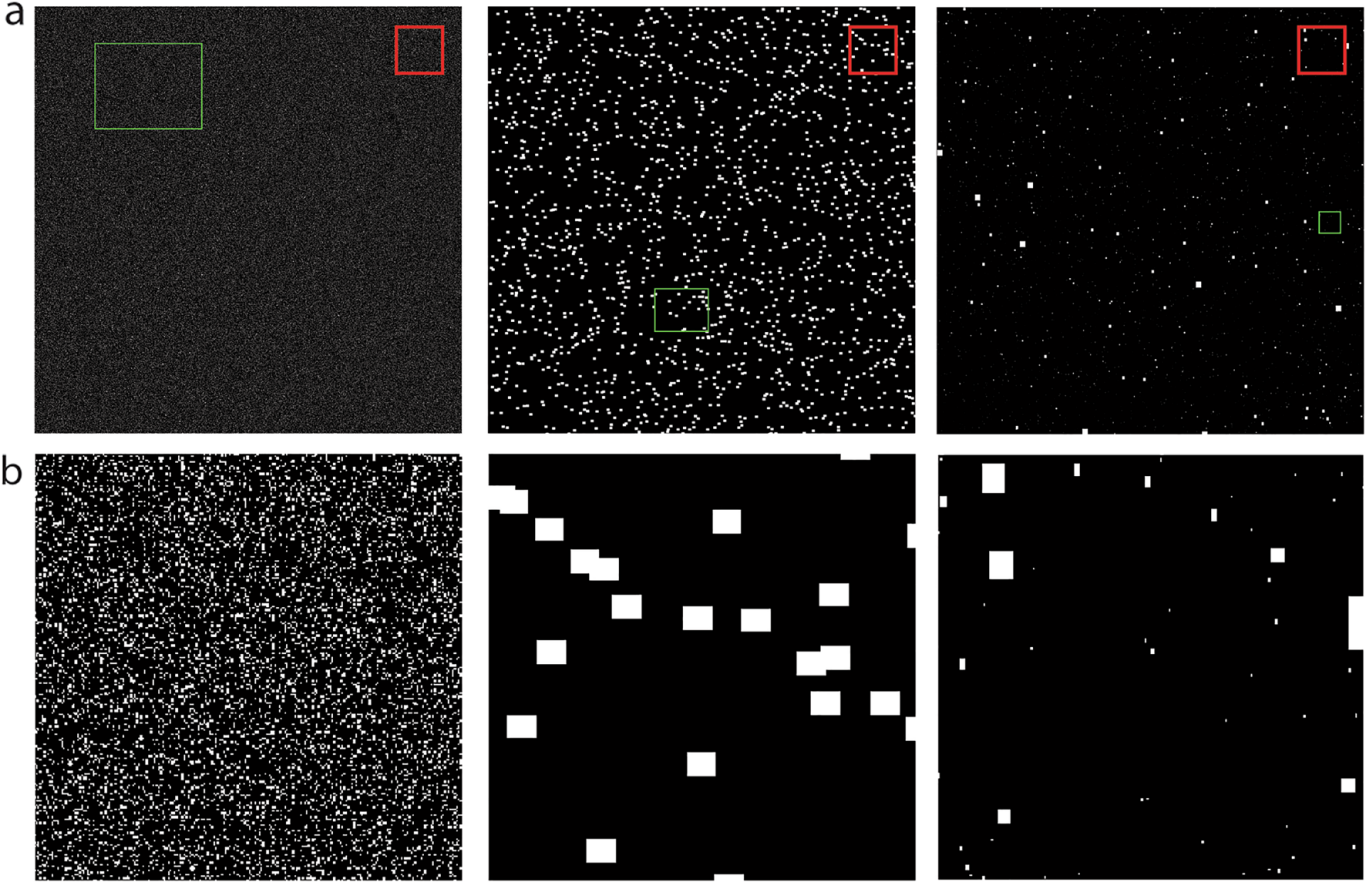
Simulated tumors by the computer-based model. **a** Simulated tumors differed by aggregates size, biopsy size and percentage of PD-L1 positivity. Left and middle—homogenous tumors, right—heterogenous tumor. Black—cells that are negative for PD-L1, white—cells that are positive for PD-L1, green—simulated biopsy, red—area magnified below. **b** Magnification of A

MATLAB software version R2017a was used to produce multiple matrices, where each matrix cell represented a typical immune cell, with a diameter of 10 µm, or calculated as an area of 100 µm^2^ as a matrix cell is a square. Each matrix represented a "*tumor*", with a dimension of 10 cm × 10 cm. Such a large "tumor" in the model was required for good representation of the different positive cell distribution, especially in cases with low PD-L1 positive cells fraction. An immune cell "expressing" PD-L1 protein received the value 1 whereas a cell that is negative for PD-L1 received the value 0. The "tumor" consisted of immune cells rather than tumor cells as VENTANA PD-L1 (SP142) assay calculate the proportion of tumor area that is occupied by PD-L1 staining tumor infiltrating immune cells. From each matrix or "*tumor*", a section was taken, to represent a "*biopsy*". A trial contained over a thousand "*tumors*" and "*biopsies*", which differed in the expression of PD-L1. The distribution of PD-L1 expression was based on our clinical finding and previous research [9]. 60% of cases had PD-L1 expression below 1%, approximately 20% of cases had PD-L1 expression between 1 and 5%, and 20% had PD-L1 expression of 5–20%. The model could either generate homogenous tumors, with the same aggregate or single cell sizes distributed in the entire tumor, or it could generate heterogenous tumors, with different sizes of aggregates, meaning each tumor had small and large aggregates in the same tumor, which were matched with the clinical sample findings (Fig. 1).

Several parameters that might lead to inaccurate results were evaluated in this model. The first is the size of the biopsy. The smaller the biopsy the less it represents the entire tumor and accordingly, may lead to increased risk for false results. The second parameter is the size of an aggregate. Aggregate represents a cluster of PDL-1 positive cells. Larger aggregates (higher number of positive PDL-1 cells adjacent to each other) might lead to more heterogeneous distribution of PD-L1 expression and hence might increase the risk for false results. Those parameters were statistically evaluated in the homogenous tumors, as each parameter could be isolated.

We ran the heterogenous modality ten times for each sample size and ran ten times each option for the homogenous modality. We received the following outputs: The percentage of expression of the PD-L1 in every tumor, the percentage of expression of the PD-L1 in every biopsy and the error rate, measured by the area under the curve. An error means discrepancies between the tumor treatment decision and the biopsy treatment decision, when the cutoff used for determining eligibility for anti-PD-L1 therapy was 1%. Additionally, false negative and false positive were measured.

A step-by-step description of the algorithm as well as the actual scripts can be observed in Supplementary material.

### Statistical analysis

In the computer-based model we examined the effect of the biopsy size and the aggregate size on the error rate, using 2-sided, nonmatched *t*-test. *P* values < 0.05 were considered statistically significant. Additionally, we examined whether closer values of PD-L1 expression to the cutoff involves increase false results using Chi-square test.

## Results

### Clinical samples

Ten surgical samples of TNBC were analyzed in this study and a single FFPE slide from each case was stained for PD-L1. All slides were stained successfully with adequate positive and negative controls. Of the ten samples, two were positive cases and eight had low PD-L1 expression (< 1%). These cases were divided to three different groups based on PD-L1 status. Three cases had less than 0.1% of PD-L1 staining, four had 0.1–0.5% PD-L1 positivity and three cases had more than 0.75% PD-L1 expression. One case had low PD-L1 expression of 0.89% while two of them were positive with up to 10.2% of PD-L1 staining.

**Fig. 2:**
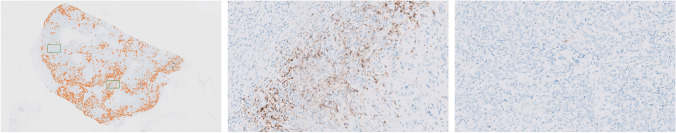
Heterogeneous distribution of PD-L1 in a representative specimen. Left—Entire specimen. Orange—aggregates annotations, green—magnified areas. Middle—high level of PD-L1 expression. Right—low level of PD-L1 expression

**Fig. 3:**
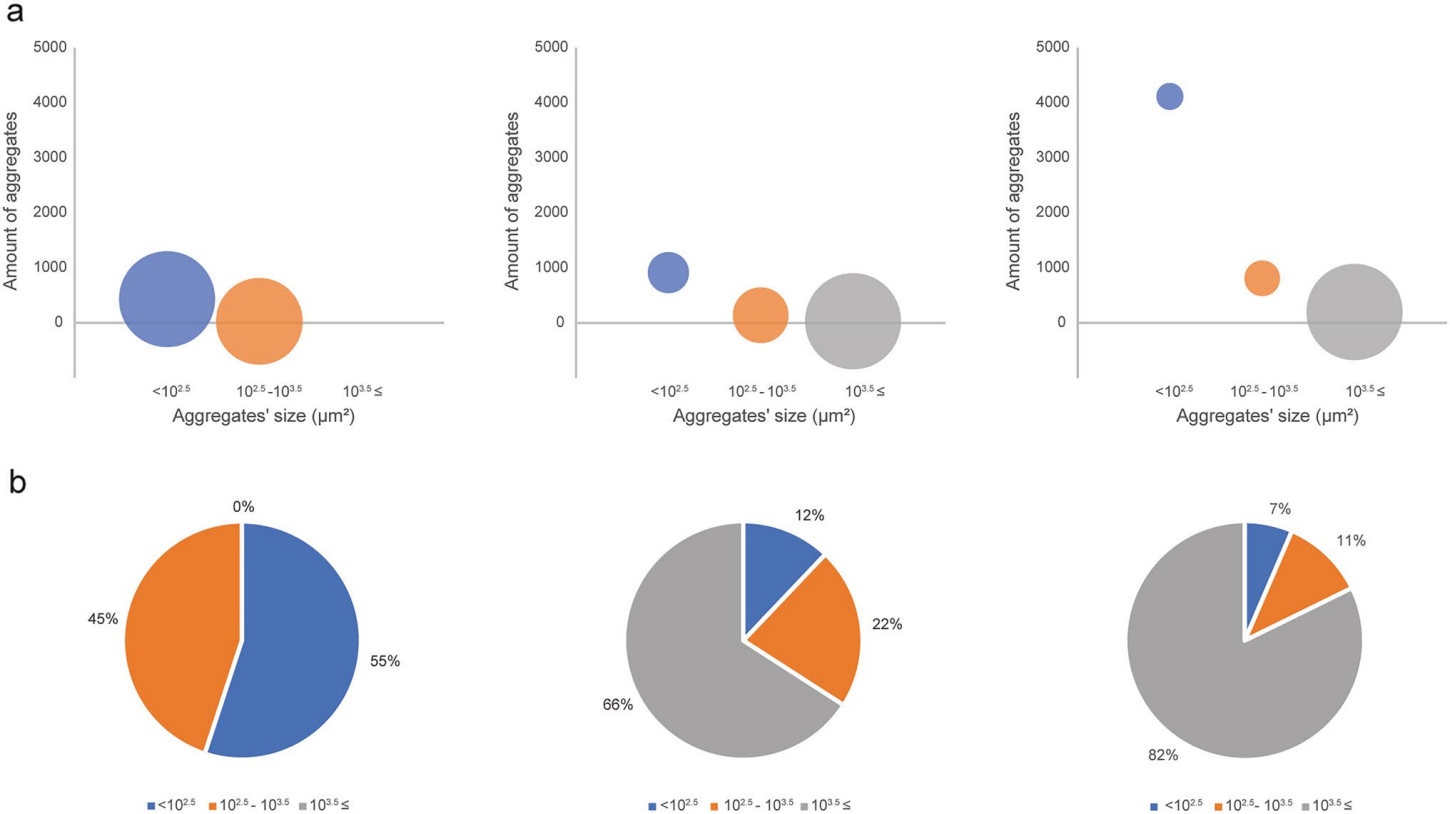
Aggregates' distribution in three groups with different PD-L1 positivity status. Left—cases with less than 0.1% of PD-L1 staining, middle- cases with 0.1–0.5% of PD-L1 staining, right—cases with more than 0.75% of PD-L1 staining. **a** The number of aggregates per aggregate size. The circles' size represents the proportion of aggregate sizes in the entire PD-L1 positive area. **b** Percentage of each aggregate size in the entire PD-L1 positive area

**Fig. 4:**
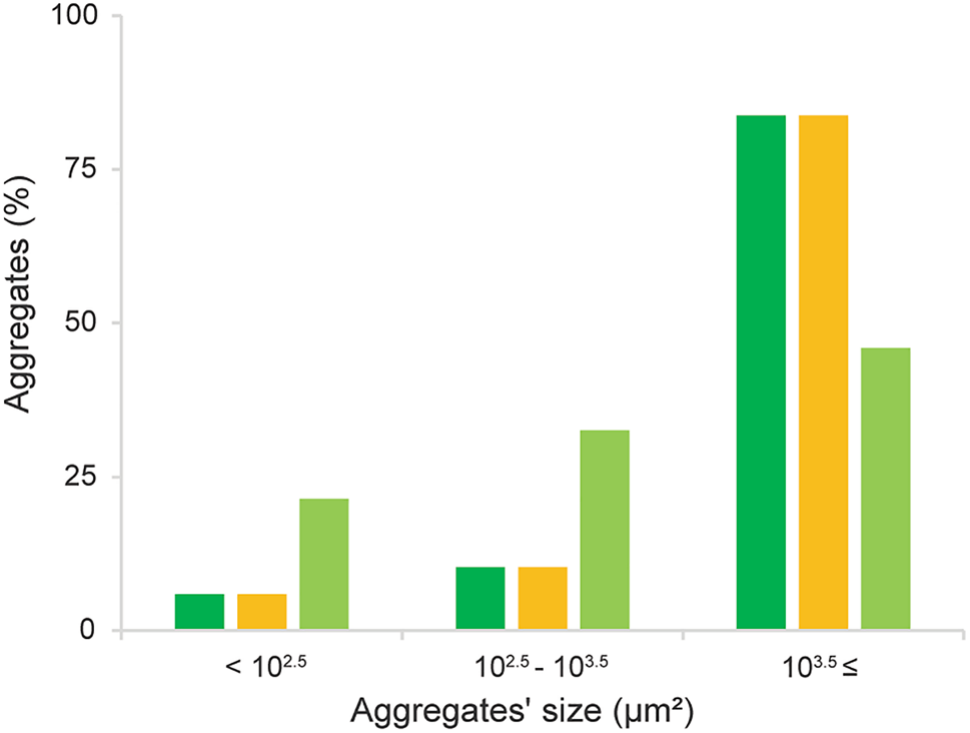
Percentage of different aggregate sizes from the total area of PD-L1 positivity in three cases with high PD-L1 positivity

Intra-tumoral heterogeneity was observed upon cases examination and further analysis of aggregates' distribution showed heterogeneity of aggregates sizes in each tumor (Figs. 2, 3). Interestingly, in tumors with less than 0.1% PD-L1 expression the largest aggregate was 0.003 mm^2^, whereas in tumors with more than 0.5% PD-L1 expression there were aggregates reaching up to 0.473 mm^2^. Furthermore, in the cases with higher PD-L1 expression, although the majority of aggregates were small, the majority of PD-L1 positive immune cells came from large aggregates (Fig. 4). A further division of PD-L1 staining can be observed in supplementary Fig. 1.

### Computer-based model

Biopsy accuracy is of paramount importance, as it guides us in choosing the most suitable treatment for TNBC. The computer-based model gave us the opportunity to examine how different parameters influence the error rate. This analysis showed us that both biopsy size and aggregate size of PD-L1 positive cells affect false results (Table 1).

**Table 1:** Error rate according to various parameters

Biopsy size (mm^2^)	1		2		5		10		20		50	
Aggregate size (µm^2^)	False positive	False negative	False positive	False negative	False positive	False negative	False positive	False negative	False positive	False negative	False positive	False negative
100	4.14	0.67	3.04	0.44	1.81	0.44	1.21	0.27	0.94	0.16	0.51	0.16
200	5.34	0.67	3.97	0.40	2.26	0.22	1.60	0.22	1.12	0.11	0.76	0.11
600	8.79	1.02	6.24	0.69	4.00	0.47	2.92	0.47	2.02	0.27	1.17	0.16
1800	13.13	2.38	9.45	1.80	6.47	0.87	5.01	0.73	3.13	0.36	2.19	0.31
4900	12.86	6.01	12.53	3.72	9.51	1.76	7.49	1.18	5.39	0.69	3.55	0.40
18,000	22.62	11.36	15.55	8.91	17.54	4.14	12.92	2.56	9.24	1.83	6.68	0.82

False positive—the error rate of all negative cases (< 1%). False negative—the error rate of all positive cases (≥ 1%)

In the homogenous tumors, the average error rate for aggregate size of 100 µm^2^, 200 µm^2^, 600 µm^2^, 1800 µm^2^, 4 00 µm^2^, and 18,000 µm^2^ were 1%, 1.2%, 2.1%, 3.4%, 4.7%, and 8%, respectively (*P* > 0.001). This finding indicate that larger aggregates would lead to higher false result rate.

**Fig. 5:**
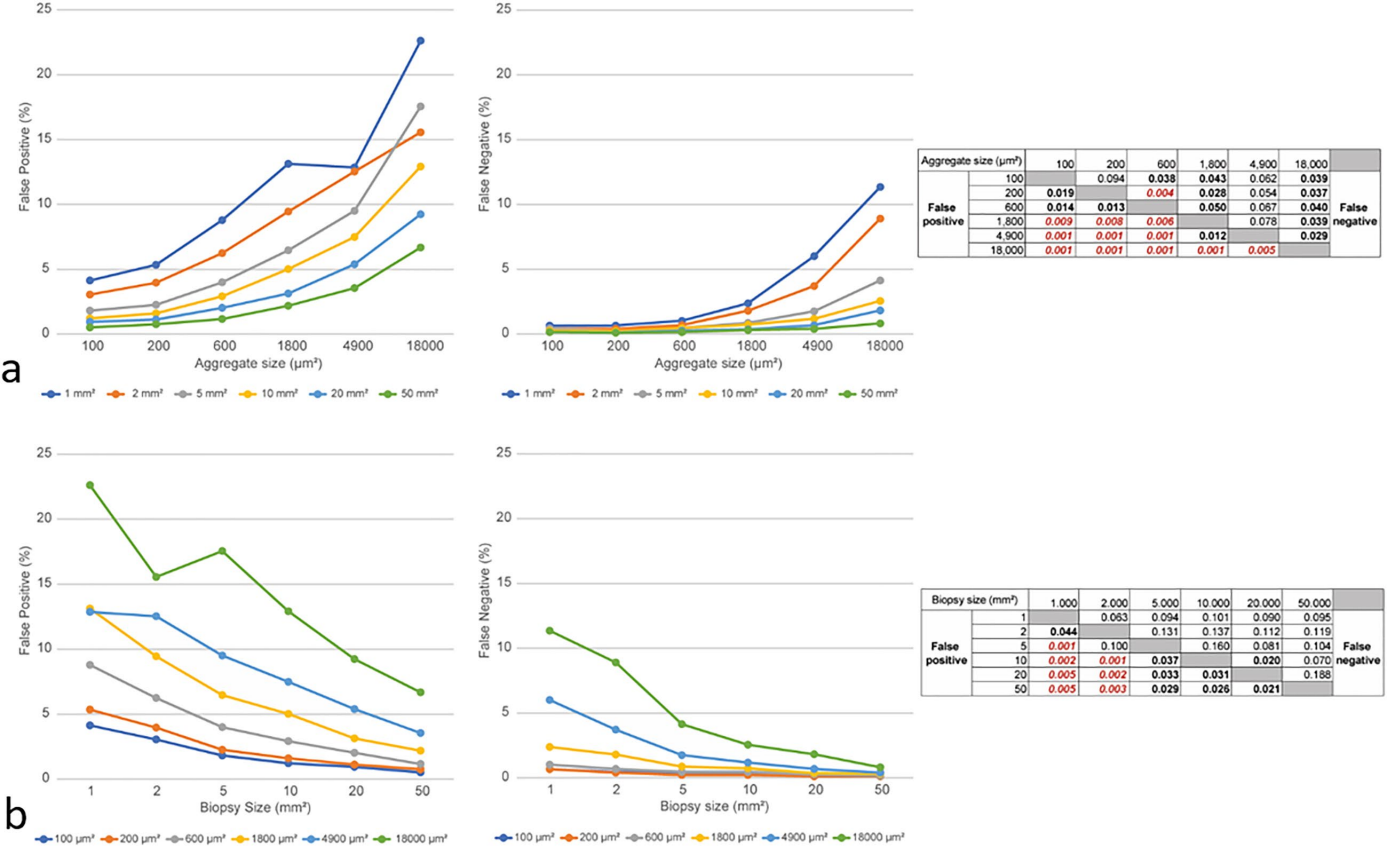
False positive and negative results based on biopsy size and aggregate size. **a** False positive (left) and false negative (middle) depending on different aggregate sizes. Each line represents specific biopsy size. Table: paired *t*-tests between different aggregate sizes. Bold—*P* < 0.05, red—*P* < 0.01. **b** False positive (left) and false negative (middle) depending on different biopsy sizes. Each line represents specific aggregate size. Table: paired *t*-test between different biopsy sizes. Bold—*P* < 0.05, red—*P* < 0.01)

The average error rate for biopsy of 1 mm^2^, 2 mm^2^, 5 mm^2^, 10 mm^2^, 20 mm^2^, and 50 mm^2^ were 6.3%, 4.7%, 3.7%. 2.7%, 1.9%, and 1.3%, respectively (*P* > 0.001). This finding is in acceptance with our hypothesis that larger samples would lead to more accurate results (Fig. 5).

**Fig. 6:**
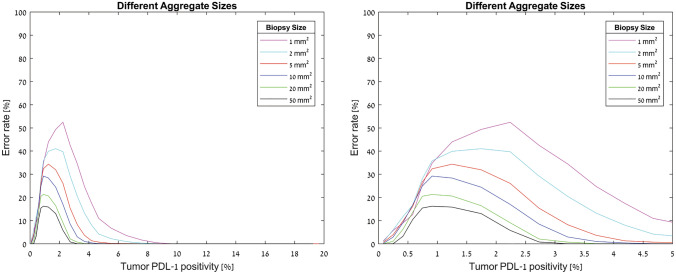
Representative graphs of computer-based model for heterogenous tumors with different biopsy sizes and different fraction of PD-L1 expression. Left- tumor PD-L1 positivity between 0 and 20%, right—tumor PD-L1 positivity between 0 and 5%

The analysis also showed that the closer the percentage of the PD-L1 expression to the cutoff value (1%), the greater the error rate. This was true across the different biopsy sizes and different aggregate sizes (Fig. 6, supplementary Fig. 2). For tumors with 0.5–1.5% positivity the error rate was 12.1% whereas the error rate for biopsies form tumors with ≤ 0.5% or ≥ 1.5% PD-L1 positivity was 0.84% and 0.65%, respectively (*P* < 0.0001, Chi-Square; Table 2).

**Table 2:** Comparison of false results between cases close to the cut-off and far from the cut-off

			Observed results in biopsies	
			True (TP and TN)	False (FP and FN)
PDL-1 positivity in tumor	Close to cut-off	Count	1,14,281	15,732
	0.5–1.5%	% within observed results	87.90%	12.10%
	Far from cut-off	Count	2,67,781	1987
	≤ 0.5% or ≥ 1.5%	% within observed results	99.26%	0.74%

Chi-square test

*TP* true positive, *TN* true negative, *FN* false positive, *FN* false negative

In our study we noticed that false negative results were significantly lower than the false positive results as false negative ranged between 0.11 and 11.36%, with an average of 1.58% while false positive ranged between 0.51 and 22.62%, with an average of 6.31% (*P* < 0.05, *t*-test).

As demonstrated in the clinical section, in real-life samples the distribution of aggregates is not homogeneous. We, therefore, used the information from the clinical samples to generate virtual samples containing different size aggregates with different quantities, better representing what we would expect in real-life samples. In the heterogeneous model, based on the clinical samples, the average error rate for biopsies of 1 mm^2^, 2 mm^2^, 5 mm^2^, 10 mm^2^, 20 mm^2^, and 50 mm^2^ were 14.23%, 13.9%, 11.4%, 10.5%, 7.9%, and 5.9%, respectively, which is a significantly higher error rate compared to homogenous tumors (Chi-square *P* < 0.001).

## Discussion

Immunotherapies such as monoclonal antibodies targeting PD-L1 and PD-1 are evolving and taking a significant role in cancer therapy in general and in breast cancer specifically [7, 9, 12]. However, it should be kept in mind that these treatments are not free from adverse events. These might include fatigue, pruritus, diarrhea, and rashes. Rarely, these therapies are related to death where the common causes are pneumonitis, pneumonia, sepsis, respiratory failure, and cardiovascular failure [13]. Therefore, finding the correct patients that will benefit from these therapies is essential, demonstrating the great importance of accurately classifying PD-L1 expression status in biopsies.

We identified a few factors that might lead to inaccurate evaluation of PD-L1 expression. The major factors include the sample size and the aggregates size. The effect of biopsy size on accurate representation of the whole tumor has been evaluated in many fields of medicine including breast cancer [14, 15]. For example, a previous study in a series of 300 breast cancer tissue showed a significant improvement in agreement between core needle biopsies and surgical excision biopsies as the biopsy size increased [15]. This is in accordance with our computer-based model that showed smaller biopsies are associated with greater false results. Additionally, our study showed that larger aggregates increase the risk for false results.

As demonstrated in the computer-based model, PD-L1 expression levels near the cutoff value were associated with higher error rate.

Interestingly, the computer-based model showed that the error rate was higher in the low PD-L1 expression cases (false positive results) compared to the positive cases (false negative results). Importantly, both false positive and false negative results can impair patients' treatment. False positive results can lead to overtreatment, adverse effects, and potential financial burden, while false negative results would make a patient not eligible for a potentially effective therapy. The frequency of these type of error should be evaluated in further studies.

Intra-tumoral heterogeneity was widely researched in non-small cell lung cancer and melanoma while only a few researches have addressed this matter in breast cancer [16–20]. Dill et al. found a discordance of 50% for tumoral PD-L1 staining of 245 breast cancer patients and 59% concordance of PD-L1 staining in the immune stroma in 91 patients. However, this research included a variety of breast cancers and did not exclusively address TNBC [21]. Additionally, Stovgaard et al. showed there was substantial heterogeneity of PD-L1 expression in 110 patients with TNBC, whereas heterogeneity was greater in immune cells rather than tumor cells [22]. Although it should be noted, Stovgaard et al. used 22C3 PD-L1 clone rather than SP142. These findings are in accordance with our clinical samples. Hence, we illustrated heterogenous tumors in our computer-based model, which demonstrated significant higher false results compared to homogenous tumors.

**Fig. 7:**
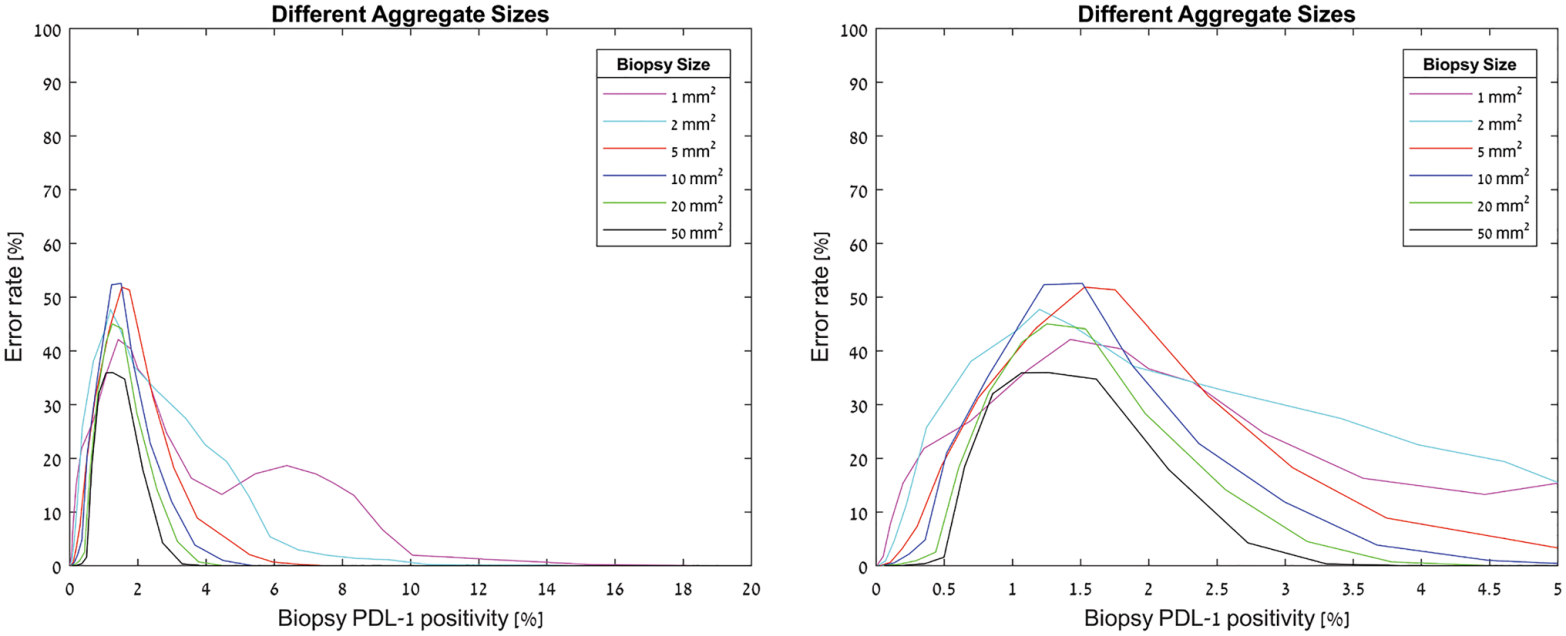
Error rate according to the fraction of PD-L1 positive cells and the biopsy size

In this study we found a few factors that affect the reliability of biopsies in representing the tumor's PD-L1 expression. Accordingly, we developed a model that can predict the risk of false results based on the fraction of PD-L1 positive cells in the biopsy and the biopsy size (Table 3, Fig. 7). As false positive cases can result in overtreatment and false negative cases miss patients that can benefit from immune checkpoint therapy, a potential clinical implication of our algorithm is to minimize these cases. Although further clinical trials are required to validate this research, our algorithm can be an addition to the oncologist toolkit that could also be taken into account for making the best treatment decision for the patient.

**Table 3:** Error rate according to biopsy size and PD-L1 positivity (%)

PD-L1 positivity in biopsies		Biopsy size (mm^2^)					
		1	2	5	10	20	50
≤ 0.1%	False negative	1.47	0.25	0.00	0.00	0.00	0.00
0.1–5%		**14.69**	8.25	3.74	1.95	0.72	0.18
0.5–1%		**36.98**	**36.96**	**17.75**	**11.37**	5.14	2.99
1–2%	False positive	**43.09**	**51.98**	**61.82**	**64.41**	**59.25**	**53.41**
2–5%		**20.58**	**27.47**	**15.82**	5.81	1.71	0.13
> 5%		6.19	1.01	0.08	0.00	0.00	0.00

Bold—high false results (> 10%)

## Supplementary Information

Below is the link to the electronic supplementary material.

Supplementary file1 (M 2 KB)

Supplementary file2 (M 1 KB)

Supplementary file3 (M 4 KB)

Supplementary file4 (M 3 KB)

Supplementary file5 (DOCX 17 KB)

Supplementary file6 (DOCX 5457 KB)

## Data Availability

The data that support the findings of this study are available from the corresponding author upon reasonable request.
