# Supplementary material for: The risk of PD-L1 expression misclassification in triple-negative breast cancer
Source: Breast Cancer Res Treat. 2022 May 27;194(2):297–305. doi: 10.1007/s10549-022-06630-3 (PMC9239943; doi:10.1007/s10549-022-06630-3)
Supplement: Supplementary file 5 — Supplementary file5 (DOCX 17 KB) [file 10549_2022_6630_MOESM5_ESM.docx]

**Matlab Code documentation**

We used two scripts to generate homogenous tumors: HomogenousTumorGeneration and createHomogeneousTumor, and two scripts to generate heterogeneous tumors: HetergeneousTumorGeneration and createHeterogeneousTumor.

The scripts **HomogenousTumorGeneration** and **HetergeneousTumorGeneration** generate multiple tumors with different PD-L1 expression by executing **createHomogeneousTumor** or **createHeterogeneousTumor** numerous times, respectively. While the first generates numerous **homogeneous** tumors, the second generates **heterogeneous** tumors.

Step-by-step of the script HomogenousTumorGeneration:

1. Definition of board or tumor size – 10X10 cm^2^ (10,000 X 10,000 cells^2^)
2. Defining aggregate shape – rectangle.
3. Defining the ideal PD-L1 expression in each generated tumor
4. A loop that generates a thousand tumors by executing the following steps:
   1. Defining the number of aggregates expressed in the tumor
   2. Generation of a tumor by executing the script createHomogenousTumor which receives as arguments the size of aggregates, the shape of aggregates defined in this script, the number of aggregates and the size of board as defined earlier.
   3. Calculating PD-L1 expression percentage.
   4. Defining a random location for biopsy/sample
   5. Creating the sample using the randomized location
   6. Defining whether the biopsy represents the same result as the tumor with a cut-off of 1% of PD-L1 expression.

- Repeating these steps until all tumors and biopsies are generated.

Step-by-step of the script createHomogenousTumor:

1. Creating the board / tumor – a matrix of zeros based on the size provided to the script.
2. Creates the shape of the aggregate according to the details provided
3. Defining random locations for all aggregates PD-L1 aggregation
4. A loop that inserts all PD-L1 aggregates according to the randomized location

- A cell that is positive for PD-L1 will be changed from zero to one.

The scripts HeterogenousTumorGeneration is similar to HomogenousTumorGeneration with the following changes:

- The script receives an array of seven different aggregate sizes instead of one.
- Defining the number of each aggregate size expressed in the tumor.
- Generation of a tumor by executing createHeterogeneousTumor.

The scripts createHeterogeneousTumor is similar to createHomogenousTumor with the following changes:

- Insertion of **different** PD-L1 aggregates sizes rather than one size.
