# Supplementary material for: The risk of PD-L1 expression misclassification in triple-negative breast cancer
Source: Breast Cancer Res Treat. 2022 May 27;194(2):297–305. doi: 10.1007/s10549-022-06630-3 (PMC9239943; doi:10.1007/s10549-022-06630-3)

**Supplementary Information**

**Supplementary figure 1**


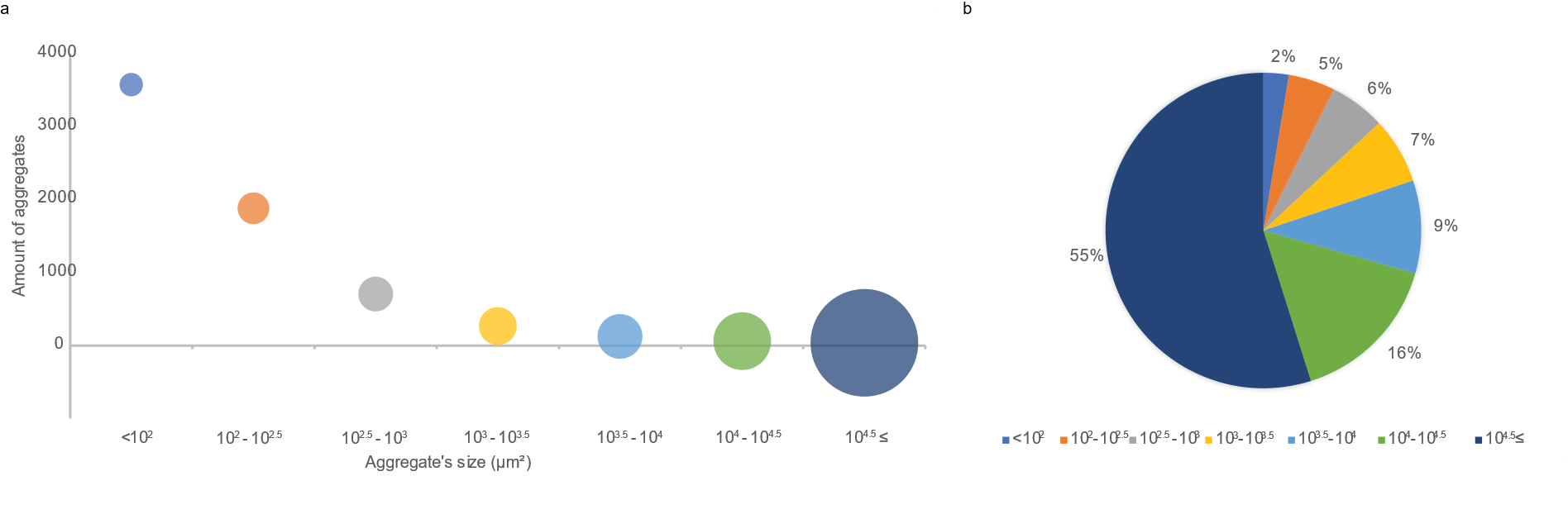
 Aggregates' distribution in the clinical samples.

Right- The number of aggregates in different aggregate sizes. The circle area represents the proportion of aggregate sizes in the entire PD-L1 positive area. Right- percentage of each aggregate size in the entire PD-L1 positive area.

**Supplementary figure 2**

Representative graphs of computer-based model for homogenous tumors with each parameter option.


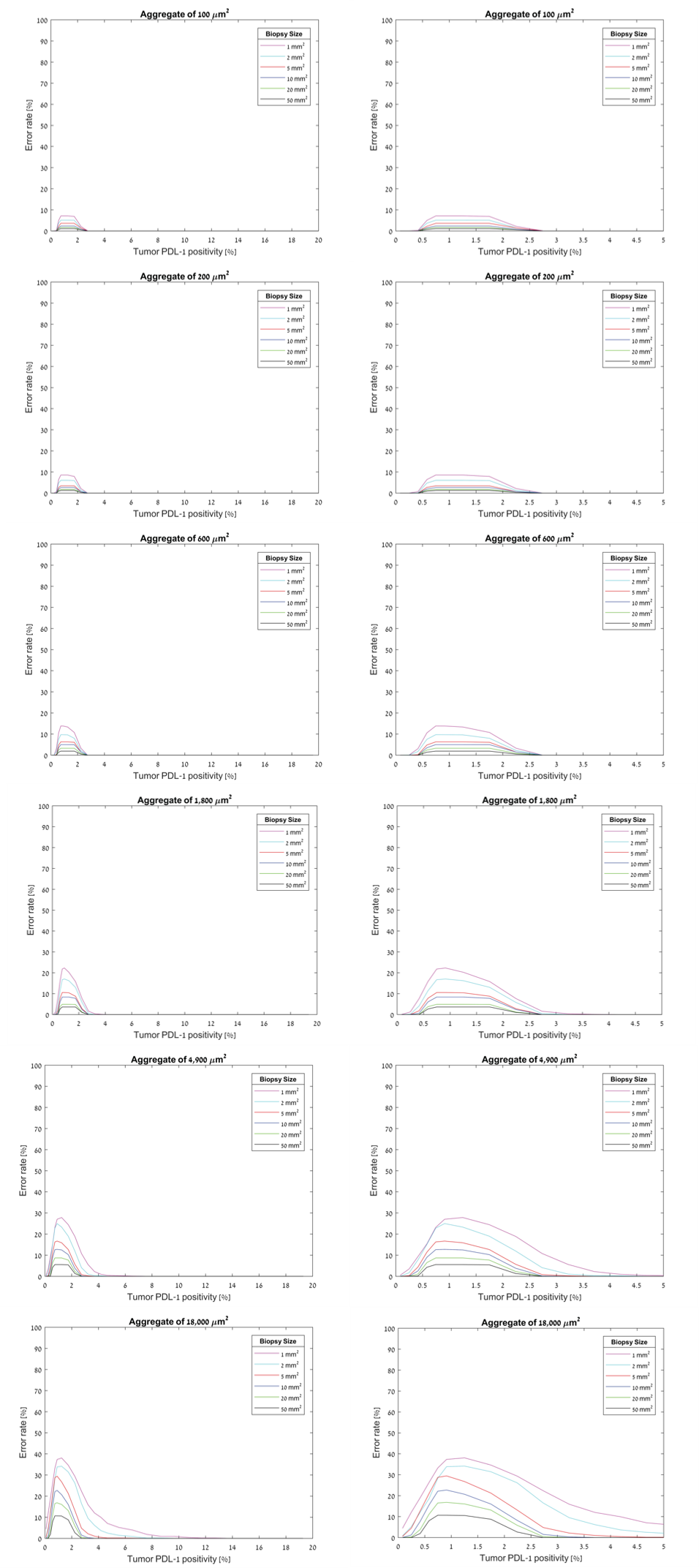


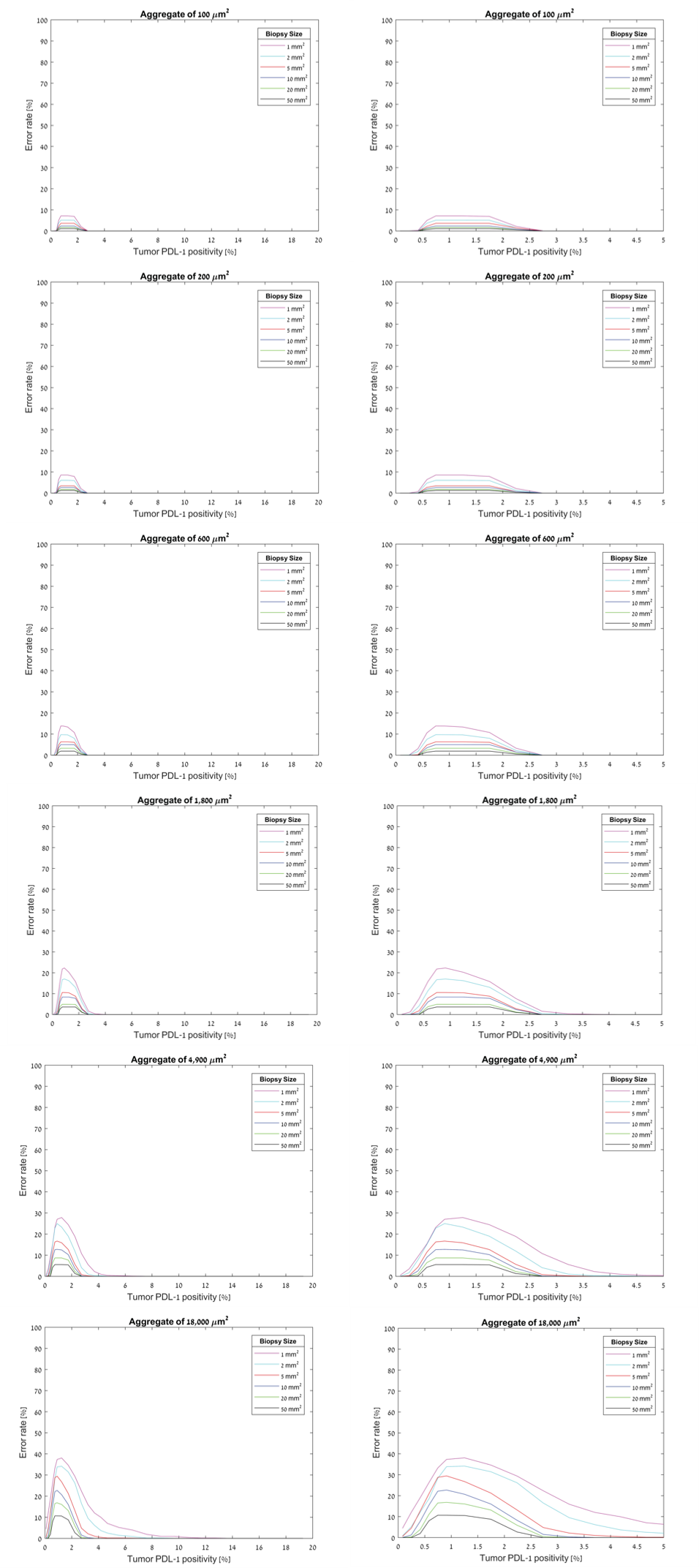


**Supplementary figure 3**


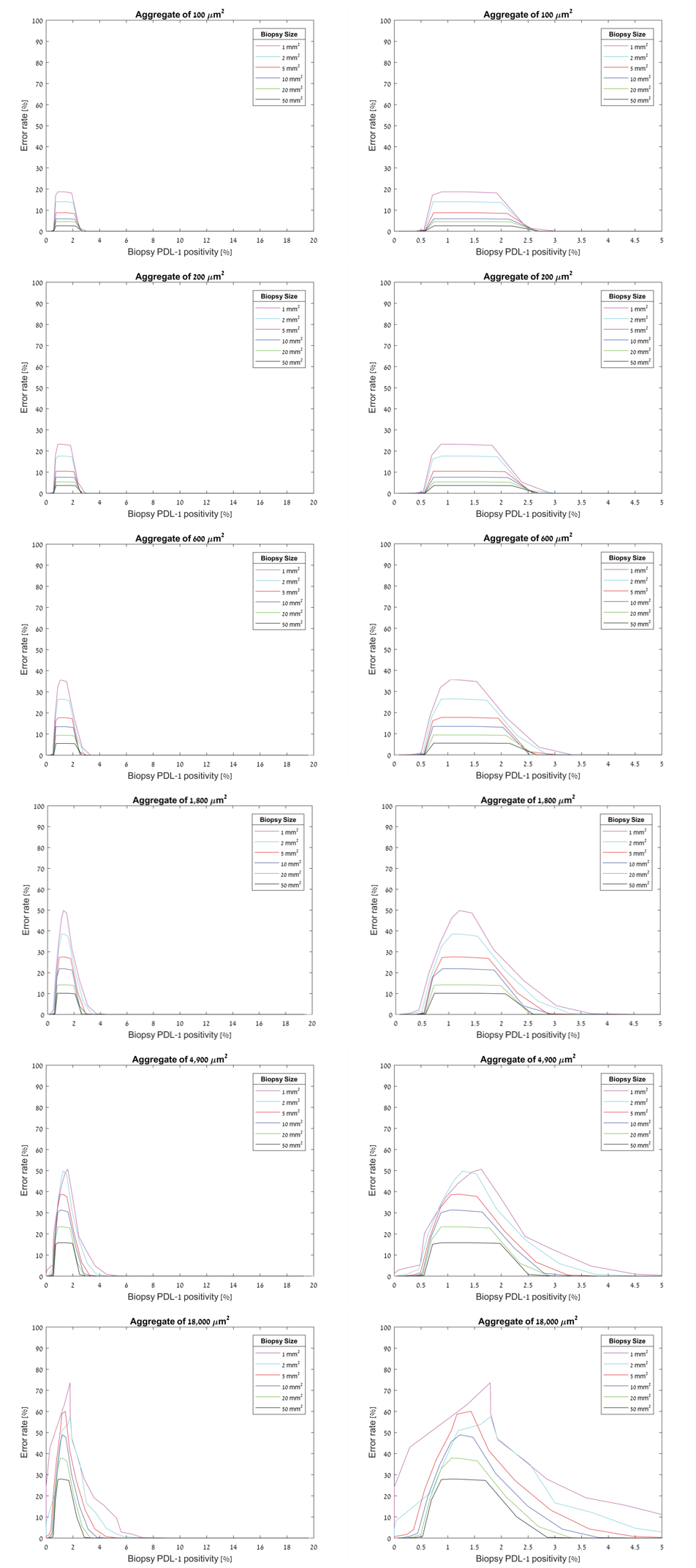
Representative graphs of computer-based model for homogenous biopsies with each parameter option.


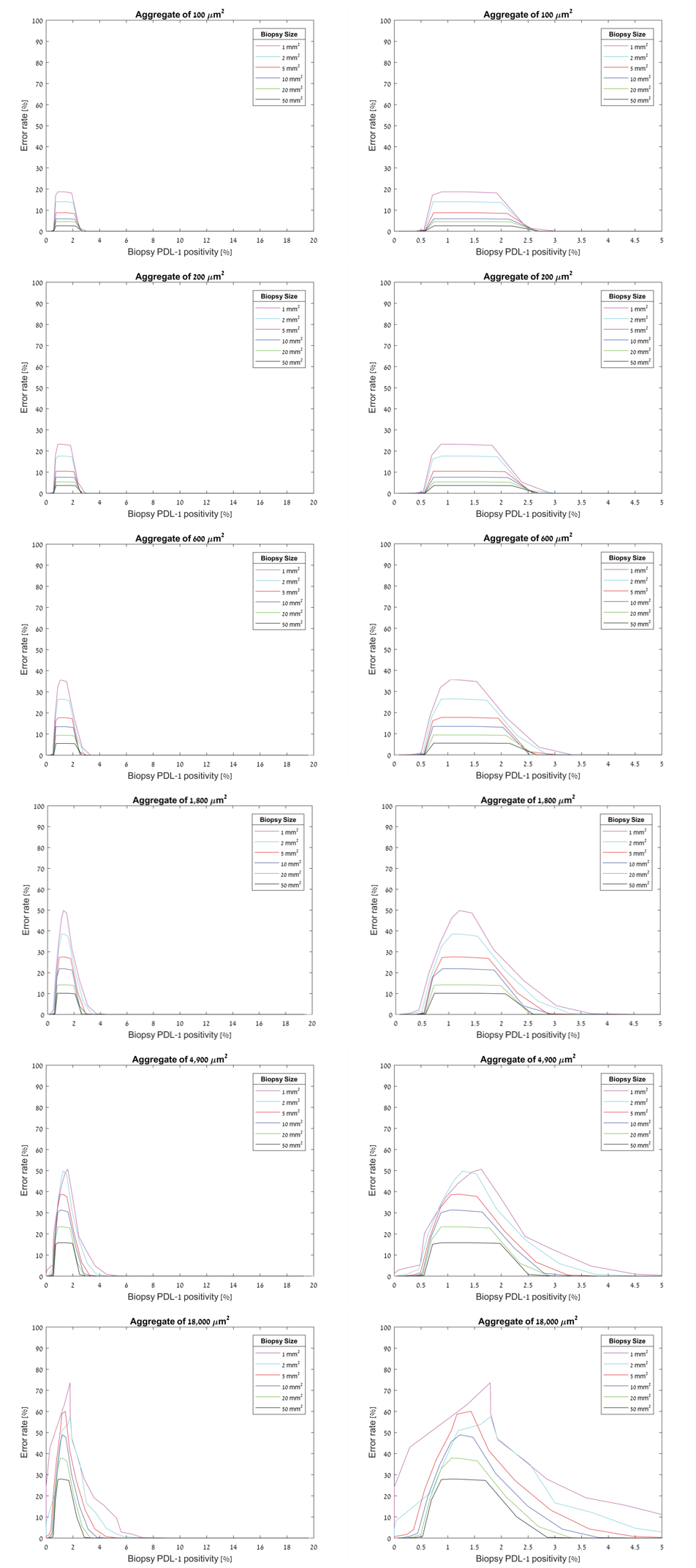

Supplement: Supplementary file 6 — Supplementary file6 (DOCX 5457 KB) [file 10549_2022_6630_MOESM6_ESM.docx]
